# Heat Shock Protein Member 8 (HSPA8) Is Involved in Porcine Reproductive and Respiratory Syndrome Virus Attachment and Internalization

**DOI:** 10.1128/spectrum.01860-21

**Published:** 2022-02-09

**Authors:** Lei Wang, Rui Li, Rui Geng, Longxiang Zhang, Xin-xin Chen, Songlin Qiao, Gaiping Zhang

**Affiliations:** a College of Veterinary Medicine, Sichuan Agricultural University, Chengdu, Sichuan, China; b Key Laboratory of Animal Immunology of the Ministry of Agriculture, Henan Provincial Key Laboratory of Animal Immunology, Henan Academy of Agricultural Sciences, Zhengzhou, Henan, China; University of Arizona

**Keywords:** PRRSV, HSPA8, GP4, attachment, internalization

## Abstract

Porcine reproductive and respiratory syndrome virus (PRRSV), a porcine arterivirus, causes severe financial losses to global swine industry. Despite much research, the molecular mechanisms of PRRSV infection remains to be fully elucidated. In the current study, we uncovered the involvement of heat shock protein member 8 (HSPA8) in PRRSV attachment and internalization during infection for the first time. In detail, HSPA8 was identified to interact with PRRSV glycoprotein 4 (GP4), a major determinant for viral cellular tropism, dependent on its carboxy-terminal peptide-binding (PB) domain. Chemical inhibitors and specific small interference RNAs (siRNAs) targeting HSPA8 significantly suppressed PRRSV infection as indicated by decreased viral RNA abundance, infectivity, and titers. Especially, PRRSV attachment was inhibited by interference of its binding to HSPA8 with mouse anti-HSPA8 polyclonal antibodies (pAbs) and recombinant soluble HSPA8 protein. HSPA8 was further shown to participate in PRRSV internalization through clathrin-dependent endocytosis (CME). Collectively, these results demonstrate that HSPA8 is important for PRRSV attachment and internalization, which is a potential target to prevent and control the viral infection.

**IMPORTANCE** PRRSV has caused huge economic losses to the pork industry around the world. Currently, safe and effective strategies are still urgently required to prevent and control PRRSV infection. As the first steps, PRRSV attachment and internalization are initiated by interactions between viral envelope proteins and host cell receptors/factors, which are not fully understood yet. Here, we identified the interaction between PRRSV GP4 and HSPA8, and demonstrated that HSPA8 was involved in PRRSV attachment and internalization. This work deepens our understanding of the molecular mechanisms involved in PRRSV infection, and provides novel insights for the development of antiviral drugs and vaccines against the virus.

## INTRODUCTION

Porcine reproductive and respiratory syndrome (PRRS) is characterized by reproductive failures and respiratory symptoms, and burdens global swine industry (1, 2). Its infectious agent, PRRSV virus (PRRSV), is an enveloped single-stranded positive-sense RNA virus and classified into the order *Nidovirales*, family *Arteriviridae* (3, 4). PRRSV infects porcine alveolar macrophages (PAMs) as primary target cells *in vivo* (5), as well as African green monkey kidney epithelial cell line MA-104 and its derivative MARC-145 *in vitro* (6). PRRSV infection is actually a complicated process, including attachment, internalization, replication, assembly, budding, and release (7). As the first steps, PRRSV attachment and internalization is initiated by interactions between viral envelope proteins and host receptors/factors on the cell surface.

PRRSV encodes several envelope proteins, such as glycoprotein (GP) 2, GP3, GP4, GP5, GP5a, membrane protein (M), and small envelope protein (E) (8). Among them, GP4 resembles a typical type I membrane protein, but does not possess a cytoplasmic tail. Importantly, PRRSV GP4 is a major determinant for viral cellular tropism (9). Previous studies have shown that GP4 interacts with CD163, an indispensable receptor for PRRSV infection (9–11). However, there is still a lack of research on its interacting proteins during PRRSV infection. In-depth identification of GP4-associated proteins will provide novel insights to develop efficient broad-spectrum vaccines and potent antiviral drugs for prevention and control of PRRS.

Heat shock protein member 8 (HSPA8) is one of the most abundant chaperones and constitutively expressed in eukaryotic cells. It is also named as heat shock cognate protein 70 (HSC70) and belongs to heat shock protein 70 (HSP70) family (12). HSPA8 contains two functional domains, an amino-terminal ATPase domain (1-383 aa, referred as AB domain) and a carboxy-terminal peptide-binding domain (393-646 aa, referred as PB domain) (13). HSPA8 plays important physiological roles in protein folding and degradation, which relies on its ATP hydrolytic activity and interactions with other cellular proteins (14). In addition, it is also reported to be involved in various stages of viral life cycle, including viral attachment (15, 16), internalization (17), and replication (18).

In this study, HSPA8 was identified to interact with PRRSV GP4 using immunoprecipitation (IP) and liquid chromatography and tandem mass spectrometry (LC-MS/MS). The role of HSPA8 in PRRSV infection was subsequently demonstrated and further analyses revealed that it was involved in PRRSV attachment and internalization during infection.

## RESULTS

### Identification of the interaction between HSPA8 and PRRSV GP4.

**FIG 1:**
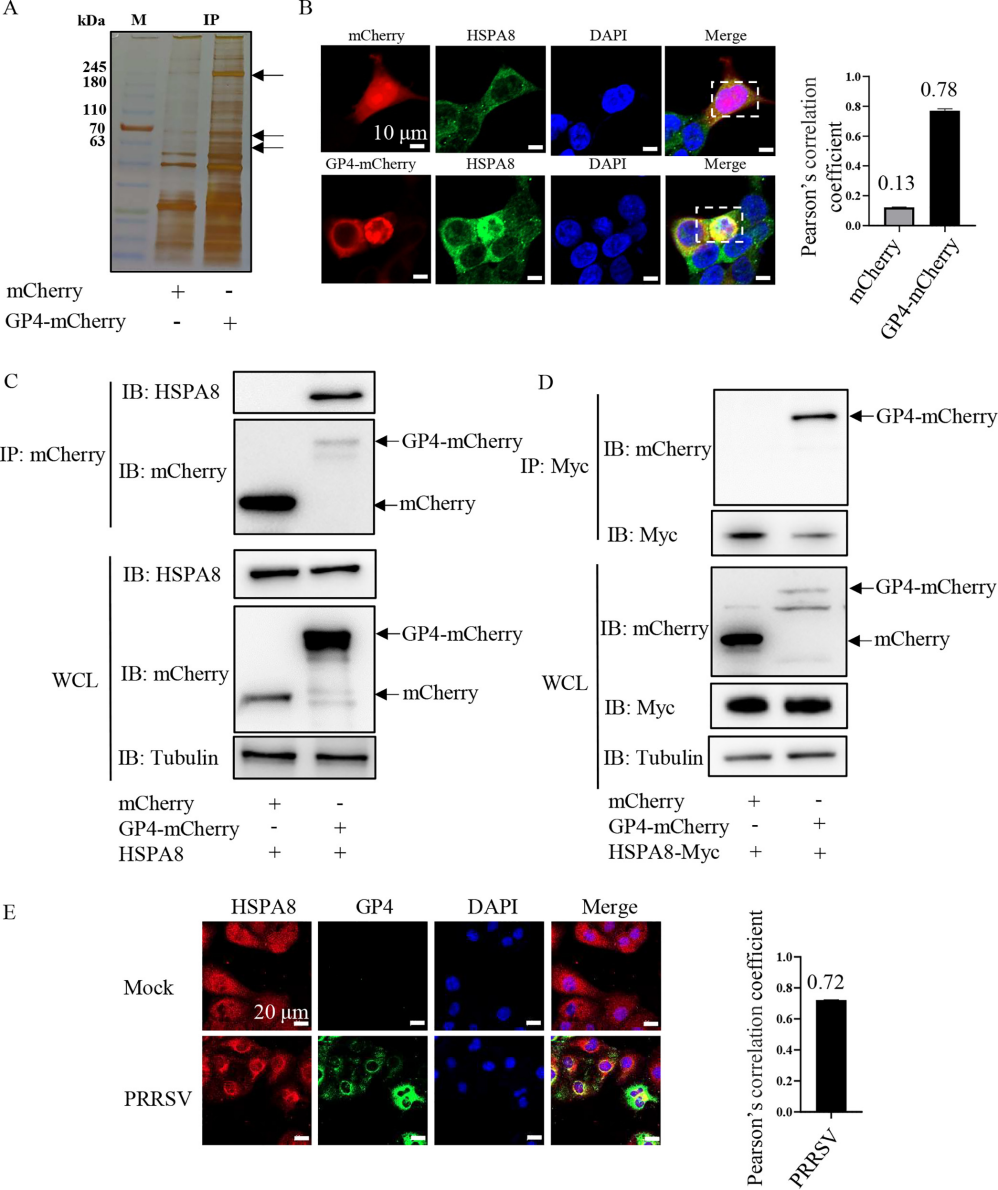
Identification of HSPA8 interacting with PRRSV GP4. (A) Silver staining of the associated proteins with GP4-mCherry. The HEK-293T cells were transfected with the plasmid expressing mCherry or GP4-mCherry. The proteins were immunoprecipitated in WCLs using anti-mCherry antibody, then separated by 12% SDS-PAGE and stained with silver. The arrows indicated different immunoprecipitated protein bands in the GP4-mCherry-expressed cells from in the mCherry-expressed ones. Lane M, protein marker. (B) GP4-mCherry co-localized with endogenous HSPA8. HEK-293T cells were transfected with the plasmid expressing GP4-mCherry (red) or mCherry (red) for 24 h, and stained with anti-HSPA8 pAbs (catalog no. 10654-1-AP; green). The cell nuclei were stained with DAPI (blue). The co-localization was assessed by determination of Pearson’s correlation coefficient. Scale bars, 10 μm. (C) GP4-mCherry interacted with endogenous HSPA8. HEK-293T cells were transfected with the plasmids expressing mCherry and GP4-mCherry, respectively. MCherry or GP4-mCherry was immunoprecipitated from WCLs by anti-mCherry antibody and their immunoprecipitated proteins were immunoblotted with anti-HSPA8 pAbs (catalog no. 10654-1-AP) and anti-mCherry pAbs. (D) GP4-mCherry interacted with exogenous HSPA8. HEK-293T cells were co-transfected with the plasmids expressing HSPA8-Myc, and mCherry or GP4-mCherry, respectively. HSPA8-Myc immunoprecipitated proteins were immunoblotted with anti-mCherry pAbs and anti-Myc MAb. (E) The endogenous co-localization between HSPA8 and PRRSV GP4 in the infected MARC-145 cells. MARC-145 cells were infected with PRRSV at 0.1 MOI for 24 h, and stained with anti-HSPA8 pAbs (catalog no. 10654-1-AP; green) and anti-GP4 pAbs (green). Nuclei were stained with DAPI (blue). The co-localization was assessed by determination of Pearson’s correlation coefficient. Scale bars, 20 μm.

As GP4 plays a critical role in PRRSV infection (9), we performed an IP assay using GP4-mCherry-expressed human embryonic kidney 293T (HEK-293T) cells to identify PRRSV GP4-interacting host cellular proteins. Silver staining indicated different immunoprecipitated protein bands in the GP4-mCherry-expressed cells from in the mCherry-expressed ones by arrows in Fig. 1A. The bands were subsequently subjected to LC-MS/MS, and the most prominent GP4-interacting proteins were non-muscle myosin heavy chain 9 (MYH9, 230 kDa), HSPA8 (70 kDa), and vimentin (54 kDa). Among them, MYH9 and vimentin have been extensively studied for PRRSV infection (19, 20). However, HSPA8 has never been reported in PRRSV infection. Therefore, we focused on HSPA8 for subsequent analyses in the current study. To corroborate the interaction between HSPA8 and PRRSV GP4, confocal microscopy was carried out and showed that endogenous HSPA8 co-localized with over-expressed GP4-mCherry but not mCherry (Fig. 1B). The co-localization coefficient between HSPA8 and GP4-mCherry was expressed as Pearson’s correlation coefficient, and the value was 0.78, suggesting that there existed an interaction (the value >0.5) (21). IP assay was further performed in the GP4-mCherry-expressed cells and confirmed their interaction (Fig. 1C). Moreover, recombinant GP4-mCherry and HSPA8-Myc were co-expressed in HEK-293T cells and IP assay showed that exogenous HSPA8 interacted with GP4 (Fig. 1D). The endogenous co-localization between HSPA8 and PRRSV GP4 was also observed in the infected MARC-145 cells by confocal microscopy (Fig. 1E). These results demonstrate that HSPA8 interacts with PRRSV GP4.

### HSPA8 PB domain is required for its interaction with PRRSV GP4 and virions.

**FIG 2:**
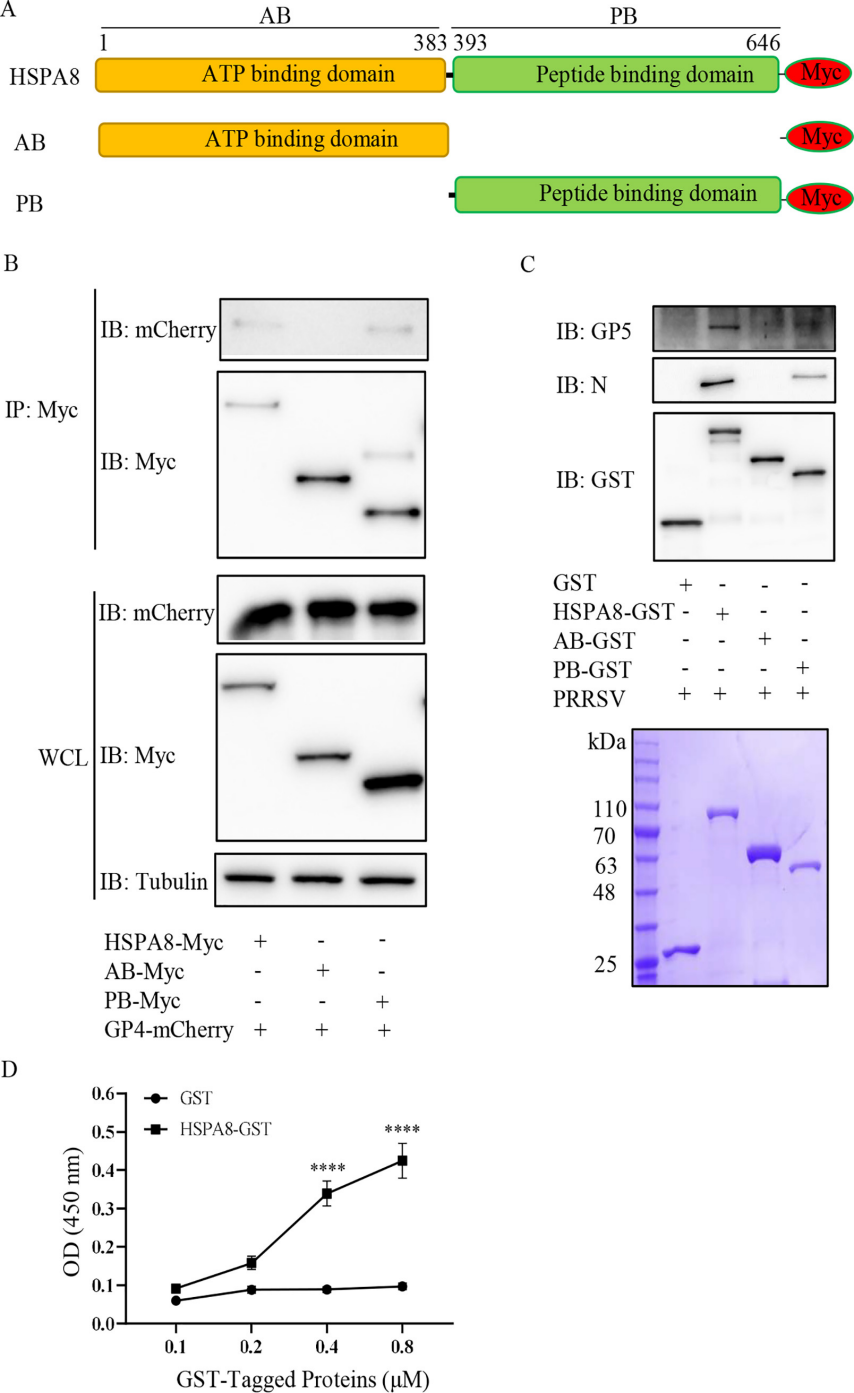
PB domain of HSPA8 interacts with GP4 and PRRSV virions. (A) The schematic diagram of HSPA8 and its truncated constructs of AB and PB domains. (B) HSPA8 PB domain interacted with GP4-mCherry. HEK-293T cells were co-transfected with the plasmids expressing GP4-mCherry and the full-length HSPA8, AB domain, or PB domain for 48 h. HSPA8-Myc, AB-Myc, or PB-Myc was immunoprecipitated from WCLs by anti-Myc MAb. The immunoprecipitated proteins were immunoblotted with anti-mCherry pAbs and anti-Myc MAb. (C, D) The recombinant HSPA8 bound to PRRSV virions. The recombinant proteins were purified by GSH beads and detected by SDS-PAGE. The purified recombinant proteins were coupled to GST beads, where GST served as control. Then the beads were incubated with PRRSV virions. The eluted samples were subjected to IB, and detected by anti-PRRSV N MAb, anti-GP5 pAbs and anti-GST MAb (C). The ELISA plates were coated with the purified recombinant GST-tagged proteins (0.1, 0.2, 0.4, 0.8 μM/well) and incubated with PRRSV virions (10^8.1^ TCID_50_/mL in PBS, 100 μL/well) at RT for 2 h. The interaction between PRRSV and HSPA8 was detected using anti-GP5 MAb and HRP-conjugated secondary antibodies to obtain the values of OD_450_. The experiments were performed in triplicate. ****, *P* < 0.0001 (D).

HSPA8 possesses AB and PB domains (Fig. 2A). To determine which domain of HSPA8 is responsible for its interaction with PRRSV GP4, we co-expressed recombinant HSPA8-Myc, AB-Myc, or PB-Myc with GP4-mCherry and then conducted IP assay. Fig. 2B showed that HSPA8 PB domain was required for its binding to PRRSV GP4. To confirm the role of HSPA8 PB domain in binding to PRRSV virions, glutathione *S*-transferase (GST) pulldown assay was performed. The recombinant HSPA8, AB, and PB domains were all successfully expressed as soluble proteins and purified by GSH beads. As shown in Fig. 2C, all these three proteins were obtained with high purity and consistent with their predicted molecular masses. We subsequently exploited the purified target proteins to pull down PRRSV virions. In parallel, GST protein was purified and used as control. HSPA8 and its PB domain were found to bind to PRRSV virions indicated by viral major envelope protein GP5 and nucleocapsid (N) protein, while its AB domain and GST protein were not (Fig. 2C). The direct interaction between HSPA8 and PRRSV virions was further confirmed by an enzyme-linked immunosorbent assay (ELISA) (Fig. 2D). These findings determine that HSPA8 PB domain is required for its interaction with PRRSV GP4 and virions.

### HSPA8 is important for PRRSV infection in MARC-145 cells.

**FIG 3:**
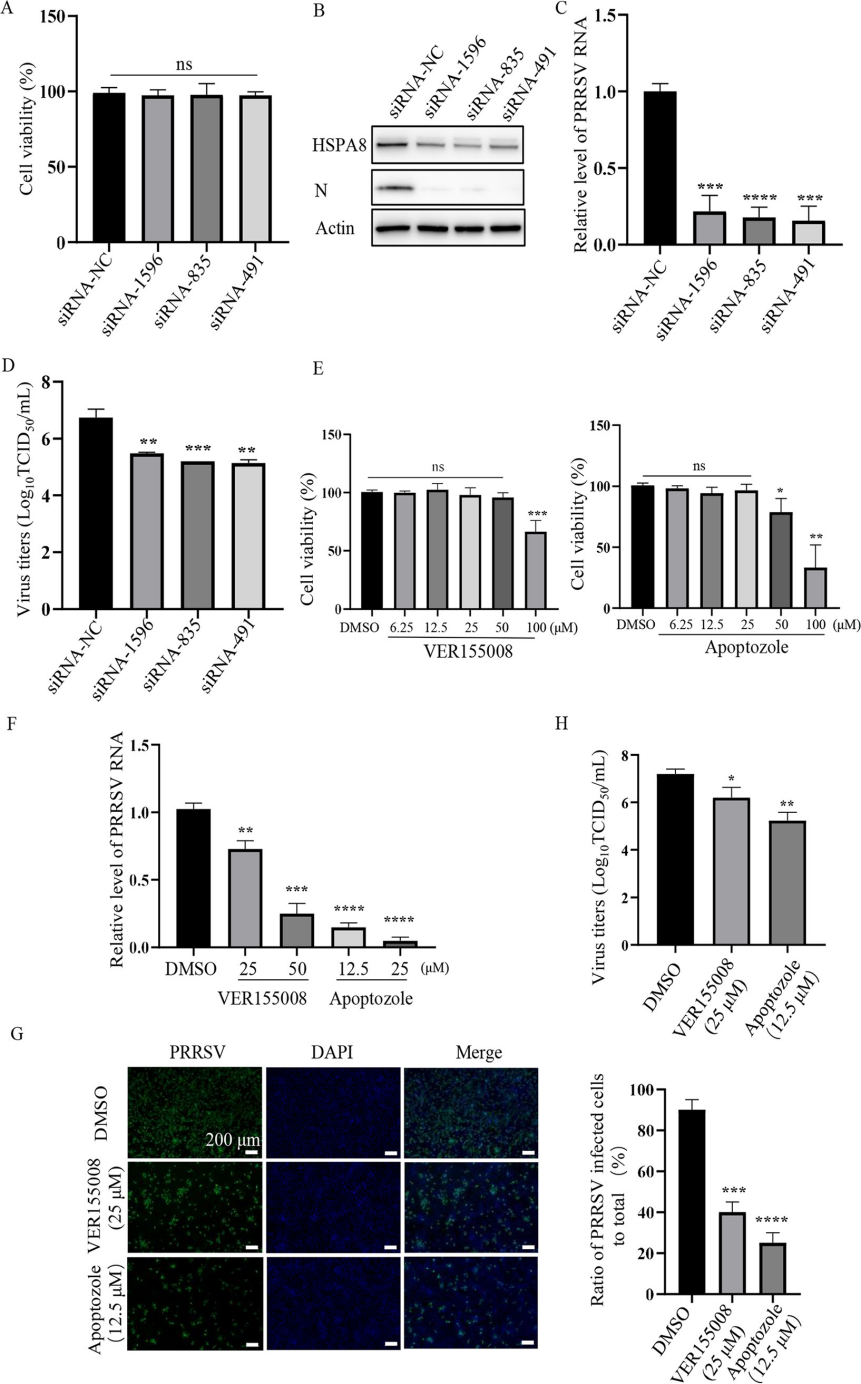
HSPA8 is important for PRRSV infection in MARC-145 cells. (A) The cyto-toxicity of siRNAs. MARC-145 cells were transfected with siRNAs against HSPA8 (no. 1596, 835 and 491) and siRNA-NC for 36 h. The transfected cells were then analyzed using the CCK-8 kit. Data represent means ± SD from three independent experiments. ns, no significant difference. (B, D) *HSPA8* knockdown decreased PRRSV infection. MARC-145 cells were transfected with siRNAs against HSPA8 (no. 1596, 835 and 491) and siRNA-NC for 36 h, and then infected with PRRSV at 0.1 MOI for 24 h. The level of HSPA8 and PRRSV N protein were determined by IB (B). PRRSV RNA abundance was determined by RT-qPCR (C), and PRRSV titers were determined by detecting TCID_50_ (D). Data represent means ± SD from three independent experiments. *, *P* < 0.05; **, *P* < 0.01; ***, *P* < 0.001; ****, *P* < 0.0001. (E) The cyto-toxicity of VER15508 and apoptozole. MARC-145 cells were treated with 6.25, 12.5, 25, 50, 100 μM VER15508 and apoptozole for 24 h. DMSO was used as control. The treated cells were then analyzed using the CCK-8 kit. Data represent means ± SD from three independent experiments. *, *P* < 0.05; **, *P* < 0.01; ***, *P* < 0.001; ns, no significant difference. (F-H) The chemical inhibitors suppressed PRRSV infection. MARC-145 cells were treated with VER155008 (25, 50 μM) or Apoptozole (12.5, 25 μM) and infected with PRRSV BJ-4 at 0.1 MOI for 24 h. PRRSV RNA abundance was determined by RT-qPCR (F). PRRSV infectivity was detected by immunofluorescence assay (IFA), and the ratio of PRRSV-infected cells to total was calculated. Scale bars, 200 μm (G). PRRSV titers were determined by detecting TCID_50_ (H). Data represent means ± SD from three independent experiments. *, *P* < 0.05; **, *P* < 0.01; ***, *P* < 0.001; ****, *P* < 0.0001.

Next, we examined the biological significance of HSPA8 during PRRSV infection in MARC-145 cells. We utilized specific small interference RNAs (siRNAs) targeting HSPA8 to detect its function. In Fig. 3A to C, non-cytotoxic *HSPA8* knockdown significantly influenced PRRSV N protein level (∼85% reduction) and RNA abundance (∼85% reduction). PRRSV progeny viral titers were also decreased by detecting 50% tissue culture infective dose (TCID_50_) in the *HSPA8* knockdown cells at 24 h postinfection (hpi; ∼1 log_10_TCID_50_/mL, Fig. 3D). These results indicate that HSPA8 is important for PRRSV infection.

We further determined whether HSPA8 ATPase activity was required for its involvement in PRRSV infection. We utilized two non-cytotoxic inhibitors VER155008 and apoptozole to inhibit HSPA8 ATPase activity (22, 23), and examined their effects on PRRSV infection (Fig. 3E). As shown in Fig. 3F, inhibition of HSPA8 ATPase by VER155008 (25, 50 μM) and apoptozole (12.5, 25 μM) significantly suppressed PRRSV replication as indicated by decreased viral RNA abundance (∼30% to 70% reduction in the VER155008-treated cells and ∼80% to 95% reduction in the apoptozole-treated ones). Subsequent PRRSV infectivity with N protein expression was impaired by about 50% and 80% at 24 hpi in the VER155008 (25 μM)- and apoptozole (12.5 μM)-treated cells, respectively (Fig. 3G). Moreover, PRRSV progeny viral titers were lowered by at least 10-fold (∼1 and ∼1.5 log_10_TCID_50_/mL in the 25 μM VER155008- and 12.5 μM apoptozole-treated cells, respectively, Fig. 3H). These data show that HSPA8 takes effect on PRRSV infection dependent on its ATP hydrolytic activity.

### HSPA8 co-localizes with PRRSV virions during attachment and internalization in MARC-145 cells.

**FIG 4:**
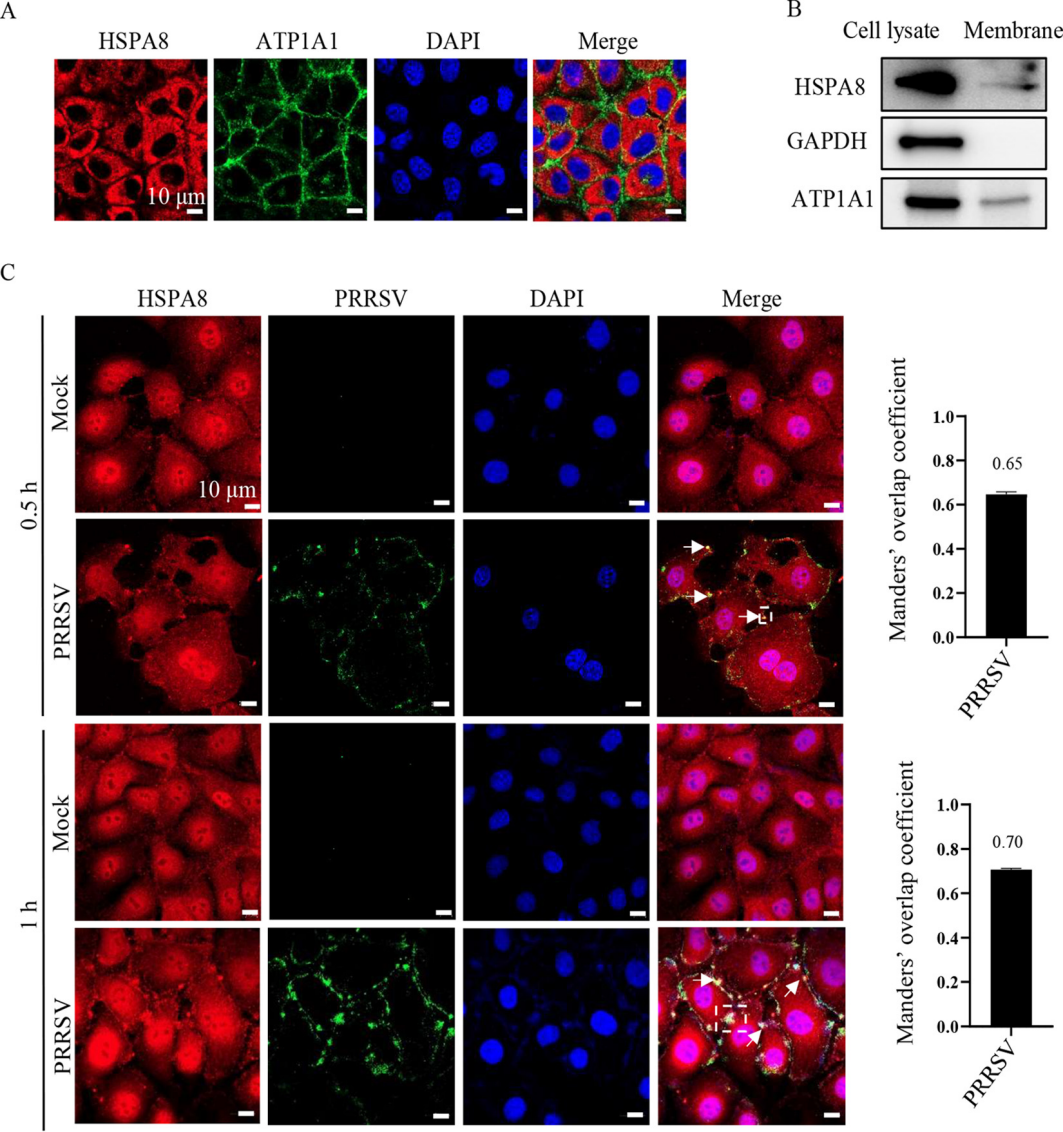
HSPA8 co-localizes with PRRSV during attachment and internalization in MARC-145 cells. (A, B) HSPA8 was expressed both on the surface and in the cytoplasm of MARC-145 cells. (A) MARC-145 cells were fixed with PFA, and stained with anti-HSPA8 MAb (catalog no. 66442-1-AP; red) and anti-ATP1A1 pAbs (green), respectively. The cell nuclei were stained with DAPI. Images were acquired on the Zeiss confocal microscope. Scale bars, 10 μm. (B) MARC-145 cell membranes were extracted. WCLs and membrane extracts were subjected to IB using anti-HSPA8 pAbs (catalog no. 10654-1-AP), anti-ATP1A1 pAbs, and anti-GAPDH pAbs, respectively. (C) PRRSV co-localized with HSPA8 in MARC-145 cells during attachment and internalization. MARC-145 cells were infected with PRRSV (10 MOI) at 37°C for 0.5 and 1 h. The cells were washed with PBS, fixed with 4% PFA, permeabilized with 0.1% Triton X-100, and stained with anti-HSPA8 pAbs (catalog no. 10654-1-AP; red) and anti-PRRSV GP5 MAb (green). The cell nuclei were stained with DAPI (blue). Images were acquired on the confocal microscope with the same confocal microscope settings. The white arrows indicated the clusters of HSPA8 with PRRSV. The Manders’ overlap coefficient in white dashed line box was analyzed. The mock-infected cells were used as control. Scale bars, 10 μm.

Based on its interaction with PRRSV GP4 and importance in viral infection, we speculated that HSPA8 probably played an important role in PRRSV early infection stage, including attachment and internalization. HSPA8 subcellular distribution was firstly monitored in MARC-145 cells by confocal microscopy. As shown in Fig. 4A, HSPA8 was found to locate both on the cell surface and in the cytoplasm of MARC-145 cells. To further confirm HSPA8 subcellular distribution, the cell membrane extract and lysates were detected by immunoblotting (IB), where Na^+^/K^+^ transporting subunit Alpha 1 (ATP1A1) was utilized as a membrane indicator (24), and glyceraldehyde-3-phosphate dehydrogenase (GAPDH) as a cell cytoplasm marker (25). HSPA8 was found both in the membrane extract and cell cytoplasm (Fig. 4B). To investigate whether HSPA8 co-localized with PRRSV virions during early infection, MARC-145 cells were inoculated with PRRSV at 37°C for 0.5 h and 1 h to allow viral attachment and internalization (26). Their co-localization was observed via confocal microscopy. In Fig. 4C, HSPA8 was homogeneously distributed in the mock-infected cells. In contrast, upon PRRSV infection at 0.5 hpi, clusters of HSPA8 were observed on the cell surface and its co-localization with PRRSV virions was evident (the value of Manders’ overlap coefficient was >0.6) (21). At 1 hpi, HSPA8 was monitored to co-localize with internalized PRRSV virions beneath the cell surface. These results show the co-localization between HSPA8 and PRRSV virions during attachment and internalization in MARC-145 cells.

### Interference with the interaction between HSPA8 and PRRSV on the cell surface inhibits viral infection in MARC-145 cells.

**FIG 5:**
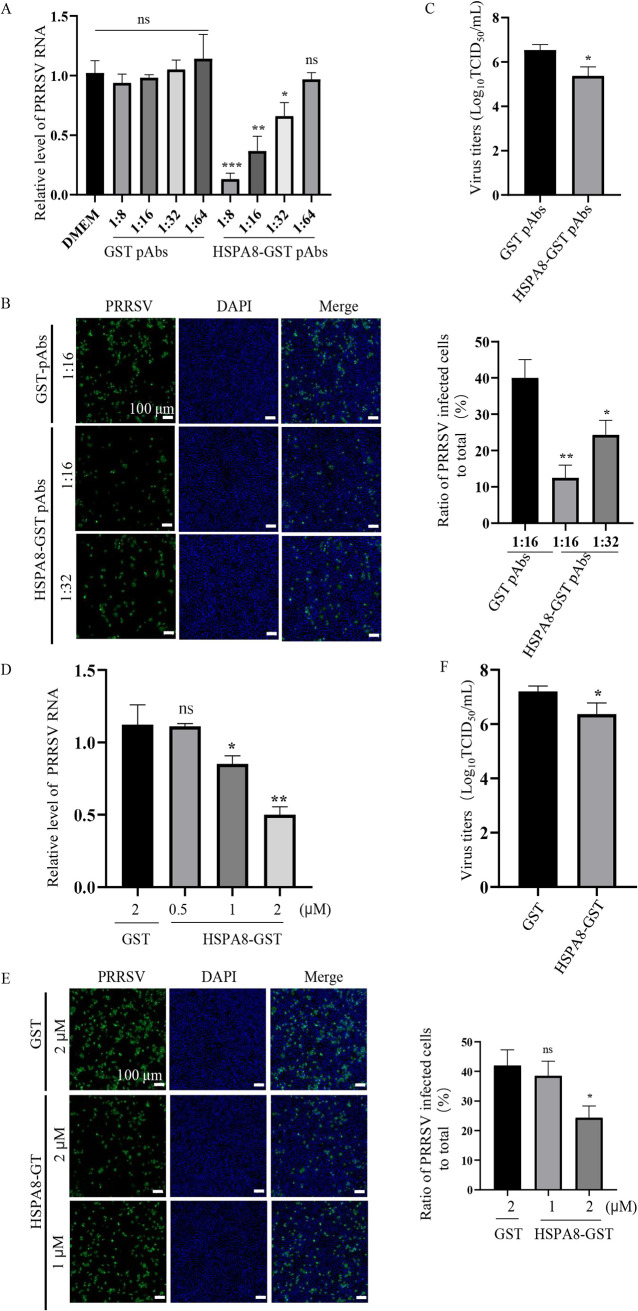
HSPA8 pAbs and soluble HSPA8 protein inhibit PRRSV infection in MARC-145 cells. (A, C) HSPA8 pAbs inhibited PRRSV infection. MARC-145 cells were incubated with different folds of diluted HSPA8-GST pAbs or GST pAbs at 37°C for 1 h. Then the cells were washed with PBS and incubated with PRRSV (0.1 MOI) at 4°C for 1 h. After three washes, the cells were again incubated with the corresponding pAbs in DMEM at 37°C for 24 h. PRRSV RNA abundance was determined by RT-qPCR (A). PRRSV infectivity was detected by IFA, and the ratio of PRRSV-infected cells to total was calculated. Scale bars, 100 μm (B). Viral titers were determined by detecting TCID_50_ (C). Data represent means ± SD from three independent experiments. *, *P* < 0.05; **, *P* < 0.01; ***, *P* < 0.001; ns, no significant difference. (D, E) Soluble HSPA8 protein inhibited PRRSV infection. PRRSV at 0.1 MOI was incubated with HSPA8-GST (0.5, 1, 2 μM) or GST protein (2 μM) at 37°C for 1 h and then inoculated in MARC-145 cells for 1 h. The cells were washed with PBS and then harvested at 24 h. PRRSV RNA abundance was determined by RT-qPCR (D). PRRSV infectivity was detected by IFA, and the ratio of PRRSV-infected cells to total was calculated. Scale bars, 100 μm (E). Viral titers were determined by detecting TCID_50_ (F). Data represent means ± SD from three independent experiments. *, *P* < 0.05; **, *P* < 0.01; ns, no significant difference.

To substantiate the role of HSPA8 in PRRSV attachment, mouse polyclonal antibodies (pAbs) against HSPA8-GST (HSPA8-GST pAbs) were prepared, and inoculated in MARC-145 cells to block the protein and interfere with its interaction with PRRSV on the cell surface. Mouse pAbs against GST (GST pAbs) were prepared and inoculated in parallel as control. Compared with medium or GST pAb treatment, HSPA8-GST pAb treatment decreased PRRSV RNA abundance in a dose-dependent manner (Fig. 5A). PRRSV infectivity was decreased by 75% and 50% in the cells inoculated with 16- and 32-fold diluted HSPA8-GST pAbs, respectively (Fig. 5B). PRRSV viral titers were also suppressed in the cells inoculated with 16-fold diluted HSPA8-GST pAbs (∼1 log_10_TCID_50_/mL, Fig. 5C).

We further addressed the importance of HSPA8 in PRRSV attachment using soluble HSPA8 protein. We mixed different doses of recombinant HSPA8-GST protein with PRRSV and then inoculated in MARC-145 cells to measure its inhibitory effect. We found that the inhibitory effect of HSPA8-GST protein was also dose-dependent, showing 50% reduction in viral RNA abundance at 2 μM (Fig. 5D). The inhibition was also demonstrated by detecting PRRSV infectivity and titers (Fig. 5E and F). These results provide evidence that HSPA8 plays a significant role in PRRSV attachment.

### HSPA8 is involved in PRRSV internalization via clathrin-dependent endocytosis in MARC-145 cells.

**FIG 6:**
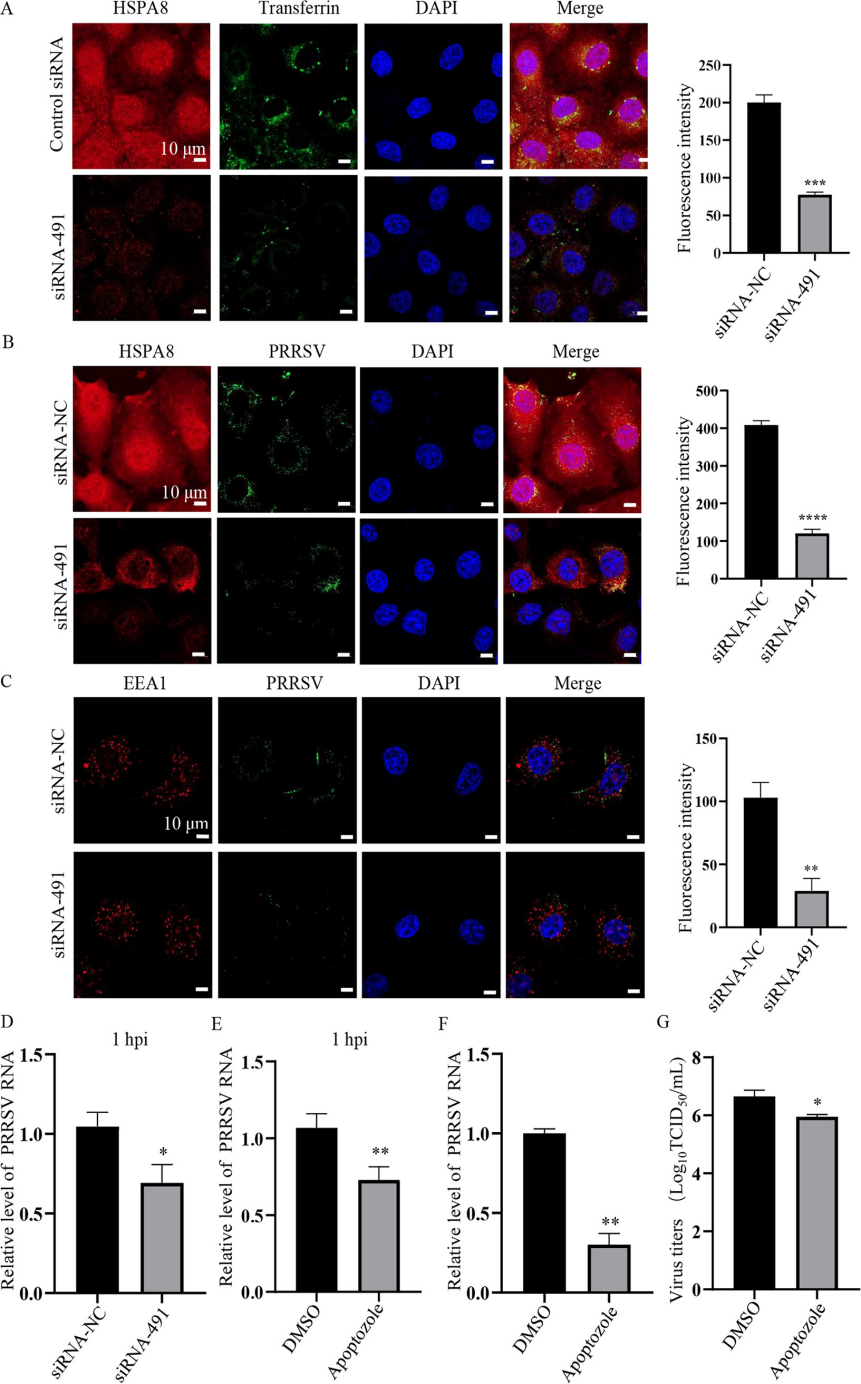
HSPA8 is involved in PRRSV internalization via CME in MARC-145 cells. (A) Knockdown of HSPA8 significantly impaired transferrin internalization in MARC-145 cells. MARC-145 cells were transfected with siRNA-NC or siRNA-491 for 36 h, and then inoculated with transferrin (5 μg/mL) at 37°C for 0.5 h. The cells were washed with CBS followed by PBS, and stained with anti-HSPA8 pAbs (catalog no. 10654-1-AP; red) and transferrin (green) antibodies. Nuclei were stained with DAPI (blue). Images were acquired on the Zeiss confocal microscope with the same confocal microscope settings. Scale bars, 10 µm. (B) Knockdown of HSPA8 significantly suppressed PRRSV internalization in MARC-145 cells. MARC-145 cells were transfected with siRNA-NC or siRNA-491 for 36 h, and then inoculated with PRRSV (10 MOI) at 37°C for 0.5 h. The cells were washed with CBS followed by PBS and stained with anti-HSPA8 pAbs (catalog no. 10654-1-AP; red) and anti-PRRSV GP5 MAb (green). Nuclei were stained with DAPI (blue). Images were acquired on the Zeiss confocal microscope, with the same confocal microscope settings. Scale bars, 10 µm. (C) The co-localization between PRRSV and EEA1 was decreased by *HSPA8* knockdown. MARC-145 cells were incubated with 10 MOI PRRSV for 0.5 h. The cells were washed with CBS followed by PBS and stained with anti-EEA1 pAbs (red) and anti-GP5 MAb (green). Cell nuclei were stained with DAPI (blue). Images were acquired on the Zeiss confocal microscope with the same confocal microscope settings. Scale bars, 10 µm. (D) Knockdown of HSPA8 significantly suppressed internalized PRRSV RNA abundance. The cells inoculated with PRRSV (10 MOI) for 1 h were harvested for PRRSV RNA abundance detection. Data represent means ± SD from three independent experiments. *, *P* < 0.05. (E, G) Apoptozole treatment impaired PRRSV internalization. MARC-145 cells were incubated with apoptozole (25 μM) and PRRSV (10 MOI) for 1 h. PRRSV RNA abundance was determined by RT-qPCR (E). MARC-145 cells were incubated with apoptozole (25 μM) and PRRSV (0.1 MOI) for 1 h, then washed and cultured with DMEM for another 24 h. PRRSV RNA abundance was determined by RT-qPCR (F). Viral titers were determined by detecting TCID_50_ at 24 h (G). Data represent means ± SD from three independent experiments. *, *P* < 0.05; **, *P* < 0.01.

HSPA8 has been reported to be involved in clathrin-dependent endocytosis (CME) (27). As PRRSV is shown to be internalized via CME (28), we hypothesize that HSPA8 also plays a role in PRRSV internalization following viral attachment. Transferrin is a marker for CME (29), and we performed confocal microscopy with transferrin in the *HSPA8* knockdown MARC-145 cells. As shown in Fig. 6A, knockdown of HSPA8 greatly influenced CME as indicated by decreased internalized transferrin. As expected, confocal microscopy showed that the internalized PRRSV virions were lowered in the *HSPA8* knockdown MARC-145 cells (Fig. 6B). A previous report has monitored that PRRSV virions are internalized into and co-localize with early endosomes marked by early endosome antigen 1 (EEA1) at 30 min postinfection (30). In Fig. 6C, *HSPA8* knockdown significantly decreased the co-localization of PRRSV and EEA1. The RNA abundance of internalized PRRSV virions was decreased by *HSPA8* knockdown as well (Fig. 6D).

To reveal whether HSPA8 ATPase activity functions in PRRSV internalization, the effect of apoptozole on PRRSV internalization was investigated. MARC-145 cells were co-inoculated with apoptozole and PRRSV for 1 h, and PRRSV internalization was decreased as shown by lowered viral RNA abundance (Fig. 6E). In parallel, the inoculated MARC-145 cells were washed, cultured, and harvested at 24 hpi for detecting viral RNA abundance and titers. PRRSV internalization was suppressed as shown by decreased RNA abundance and viral titers (Fig. 6F and G). Taken together, these results illustrate that HSPA8 is involved in PRRSV internalization via CME.

### HSPA8 is involved in PRRSV attachment and internalization into CRL-2843-CD163 cells.

**FIG 7:**
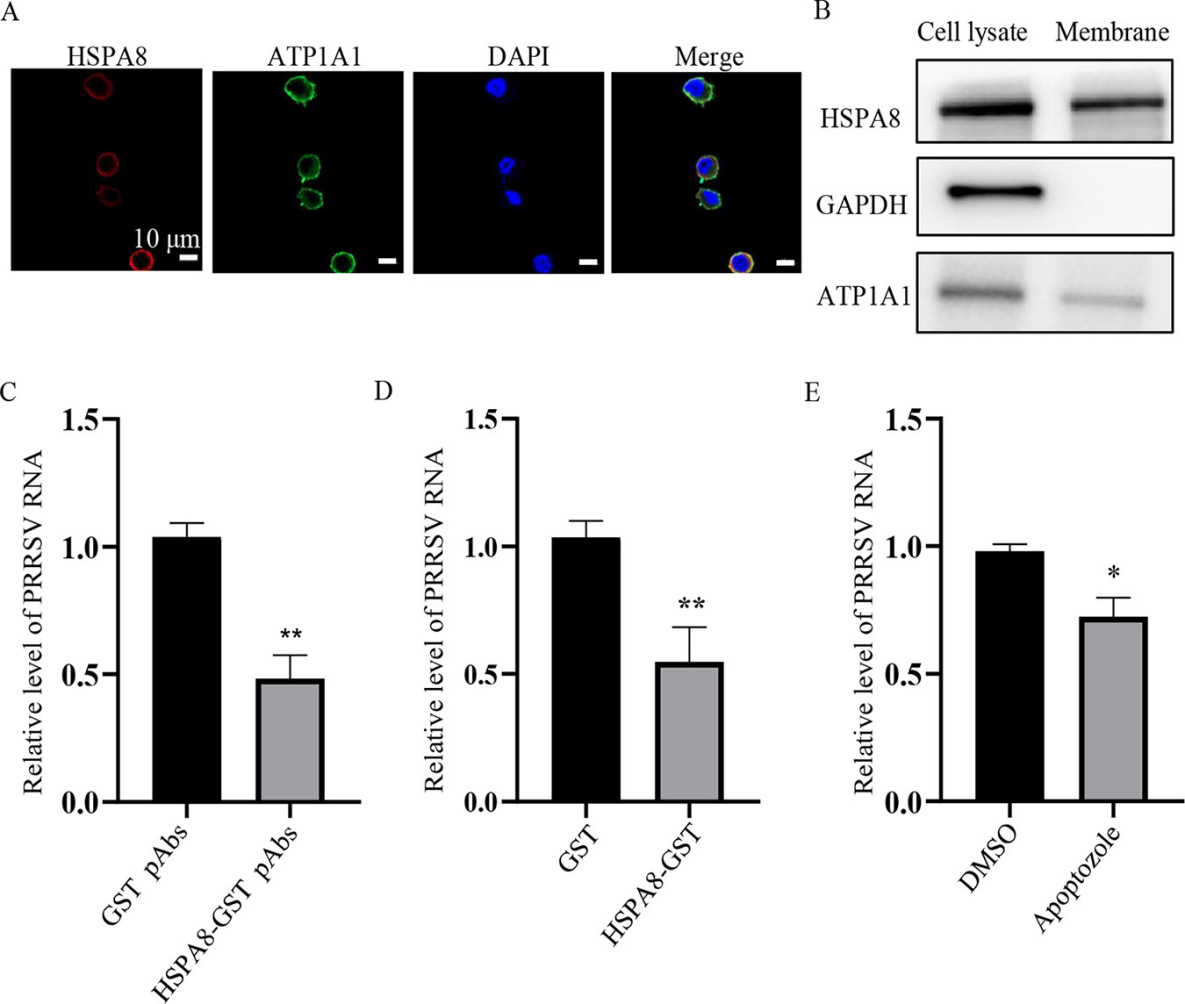
HSPA8 is important for PRRSV attachment and internalization in CRL-2843-CD163 cells. (A, B) HSPA8 was expressed both on the cell surface and in the cytoplasm of CRL-2843-CD163 cells. CRL-2843-CD163 cells were fixed with 4% PFA, stained with anti-HSPA8 MAb (catalog no. 66442-1-AP; red) and anti-ATP1A1 pAbs (green), respectively. Cell nuclei were stained with DAPI (blue) by confocal microscopy. Scale bars, 10 μm (A). WCLs and membrane extracts of CRL-2843-CD163 were subjected to IB using anti-HSPA8 pAbs (catalog no. 10654-1-AP), anti-ATP1A1 pAbs and anti-GAPDH MAb (B). (C) HSPA8 pAbs inhibited PRRSV infection. CRL-2843-CD163 cells were incubated with HSPA8-GST pAbs and GST pAbs at 1:16 dilution in DMEM at 37°C for 1 h. Then the cells were washed with PBS and inoculated with PRRSV (0.3 MOI) at 4°C for 1 h. After three washes, the cells were again incubated with DMEM containing the corresponding antibodies for 24 h. PRRSV RNA abundance was determined by RT-qPCR. Data represent means ± SD from three independent experiments. **, *P* < 0.01. (D) Soluble HSPA8 protein inhibited PRRSV infection. PRRSV at 0.3 MOI was incubated with HSPA8-GST or GST protein at the final concentration of 2 μM at 37°C for 1 h, and then inoculated in CRL-2843-CD163 cells and harvested at 24 h. PRRSV RNA abundance was determined by RT-qPCR. Data represent means ± SD from three independent experiments. **, *P* < 0.01. (E) HSPA8 inhibitor suppressed PRRSV infection. CRL-2843-CD163 cells were treated with apoptozole (25 μM) and infected with PRRSV at 0.3 MOI for 1 h. After three washes, the cells were again cultured for 24 h. PRRSV RNA abundance were determined by RT-qPCR. Data represent means ± SD from three independent experiments. *, *P* < 0.05.

As PAMs are primary *in vivo* target cells for PRRSV (5), we determined whether HSPA8 was involved in PRRSV attachment and internalization into a PAM continuous cell line, CRL-2843-CD163. CRL-2843-CD163 cells stably express CD163 in CRL-2843 (derived from PAMs) and are susceptible to PRRSV infection (31). We observed that HSPA8 was expressed on the surface and in the cytoplasm of CRL-2843-CD163 cells (Fig. 7A and B). Subsequently, we determined the effects of interference with the interaction between HSPA8 and PRRSV on the cell surface of CRL-2843-CD163 cells with HSPA8-GST pAbs and soluble HSPA8 protein. As shown in Fig. 7C and D, both HSPA8-GST pAbs and soluble HSPA8 protein played a significant inhibitory effect on PRRSV RNA abundance. The impact of apoptozole on viral abundance was also demonstrated (Fig. 7E). These results show that HSPA8 is involved in PRRSV attachment and internalization in both CRL-2843-CD163 and MARC-145 cells.

### HSPA8 is involved in PRRSV-1 attachment and internalization.

**FIG 8:**
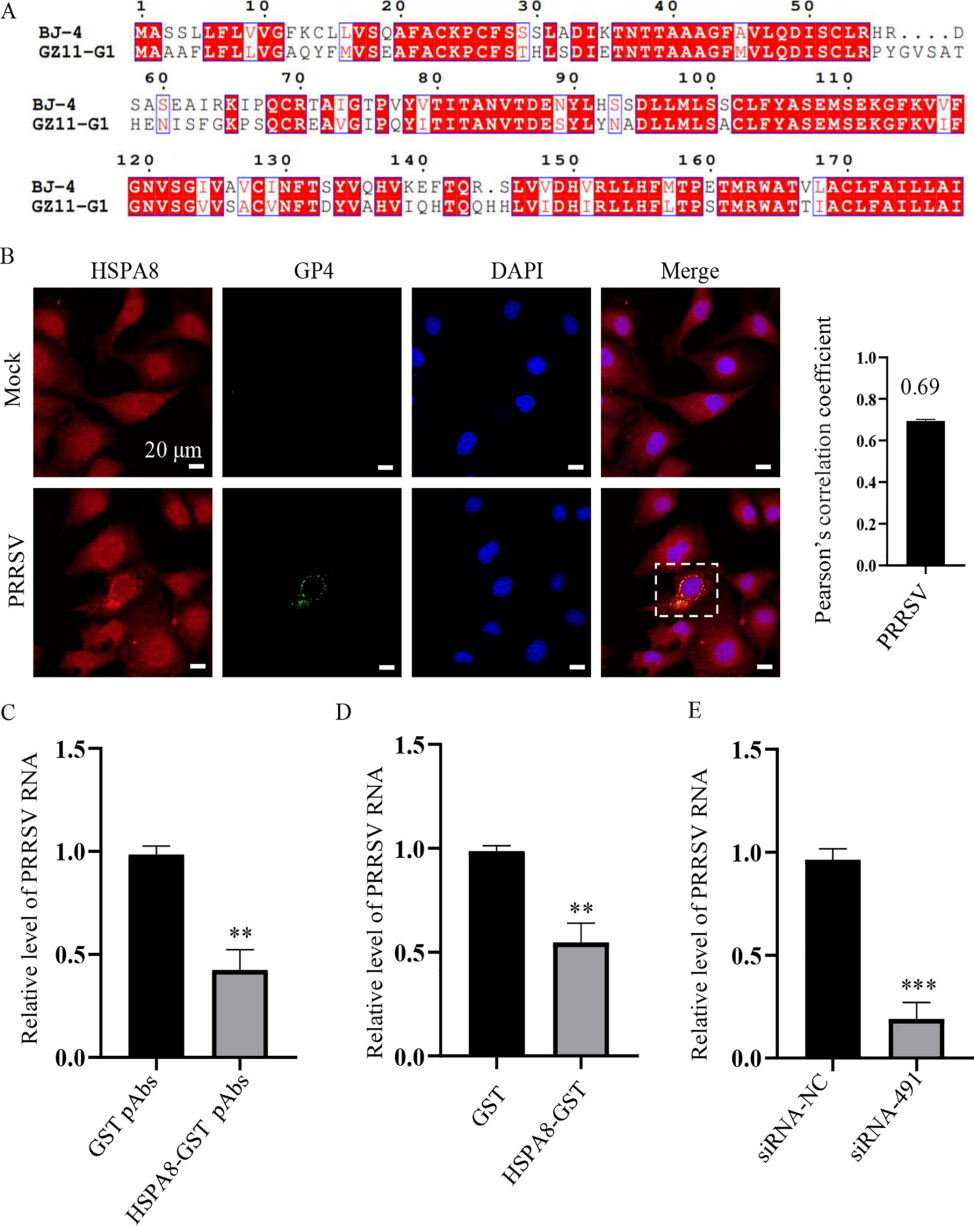
HSPA8 is important for PRRSV-1 attachment and internalization. (A) Amino acid sequence alignment of GP4 from PRRSV-1 strain GZ11-G1 (GenBank: KF001144) and PRRSV-2 strain BJ-4 (GenBank: AF331831). ClustalW and ENDscript/ESPript software were used for comparative analysis. (B) PRRSV-1 GZ11-G1 co-localized with HSPA8 in MARC-145 cells during early infection. MARC-145 cells were infected with PRRSV-1 GZ11-G1 (1 MOI) at 37°C for 24 h. The cells were washed with PBS, fixed with 4% PFA, permeabilized with 0.1% Triton X-100, and stained with anti-HSPA8 pAbs (catalog no. 10654-1-AP; red) and anti-PRRSV GP4 pAbs (green). The cell nuclei were stained with DAPI (blue). Images were acquired on the Zeiss confocal microscope. The Pearson’s overlap coefficient was analyzed. The mock-infected cells were used as control. Scale bars, 10 μm. (C) HSPA8 pAbs inhibited PRRSV-1 GZ11-G1 infection. MARC-145 cells were incubated with HSPA8-GST pAbs and GST pAbs at 1:16 dilution in DMEM at 37°C for 1 h. Then the cells were washed with PBS and inoculated with PRRSV-1 GZ11-G1 (1 MOI) at 4°C for 1 h. After three washes, the cells were again incubated with DMEM containing the corresponding antibodies for 24 h. PRRSV RNA abundance was determined by RT-qPCR. Data represent means ± SD from three independent experiments. **, *P* < 0.01. (D) Soluble HSPA8 protein inhibited PRRSV-1 infection. PRRSV-1 GZ11-G1 at 1 MOI was incubated with HSPA8-GST or GST protein at the final concentration of 2 μM at 37°C for 1 h and then inoculated in MARC-145 cells and harvested at 24 h. PRRSV RNA abundance was determined by RT-qPCR. Data represent means ± SD from three independent experiments. **, *P* < 0.01. (E) *HSPA8* knockdown decreased PRRSV-1 GZ11-G1 infection. MARC-145 cells were transfected with siRNA-491 and siRNA-NC for 36 h, and then infected with PRRSV-1 GZ11-G1 at 1 MOI for 24 h. PRRSV RNA abundance was determined by RT-qPCR. Data represent means ± SD from three independent experiments. ***, *P* < 0.001.

PRRSV isolates are divided into two genotypes: PRRSV-1 and PRRSV-2 (32). As the PRRSV strain used above was PRRSV-2 strain BJ-4, we next investigated the role of HSPA8 on PRRSV-1 strain GZ11-G1 infection. We initially aligned the sequences of GP4 from PRRSV-2 strain BJ-4 and PRRSV-1 strain GZ11-G1, and found that they shared highly conserved amino acid sequences (Fig. 8A), suggesting that HSPA8 could interact with PRRSV-1 GP4. We actually observed the endogenous co-localization between HSPA8 and PRRSV GZ11-G1 GP4 in the infected MARC-145 cells (Fig. 8B). Subsequently, we demonstrated the significant inhibitory effects of HSPA8-GST pAbs and soluble HSP8A protein on PRRSV-1 GZ11-G1 RNA abundance (Fig. 8C and D). The impact of siRNA against HSPA8 on the viral abundance was also determined (Fig. 8E). These results show that HSPA8 is involved both PRRSV-1 and PRRSV-2 attachment and internalization.

### HSPA8 contributes to PRRSV infection along with CD163.

**FIG 9:**
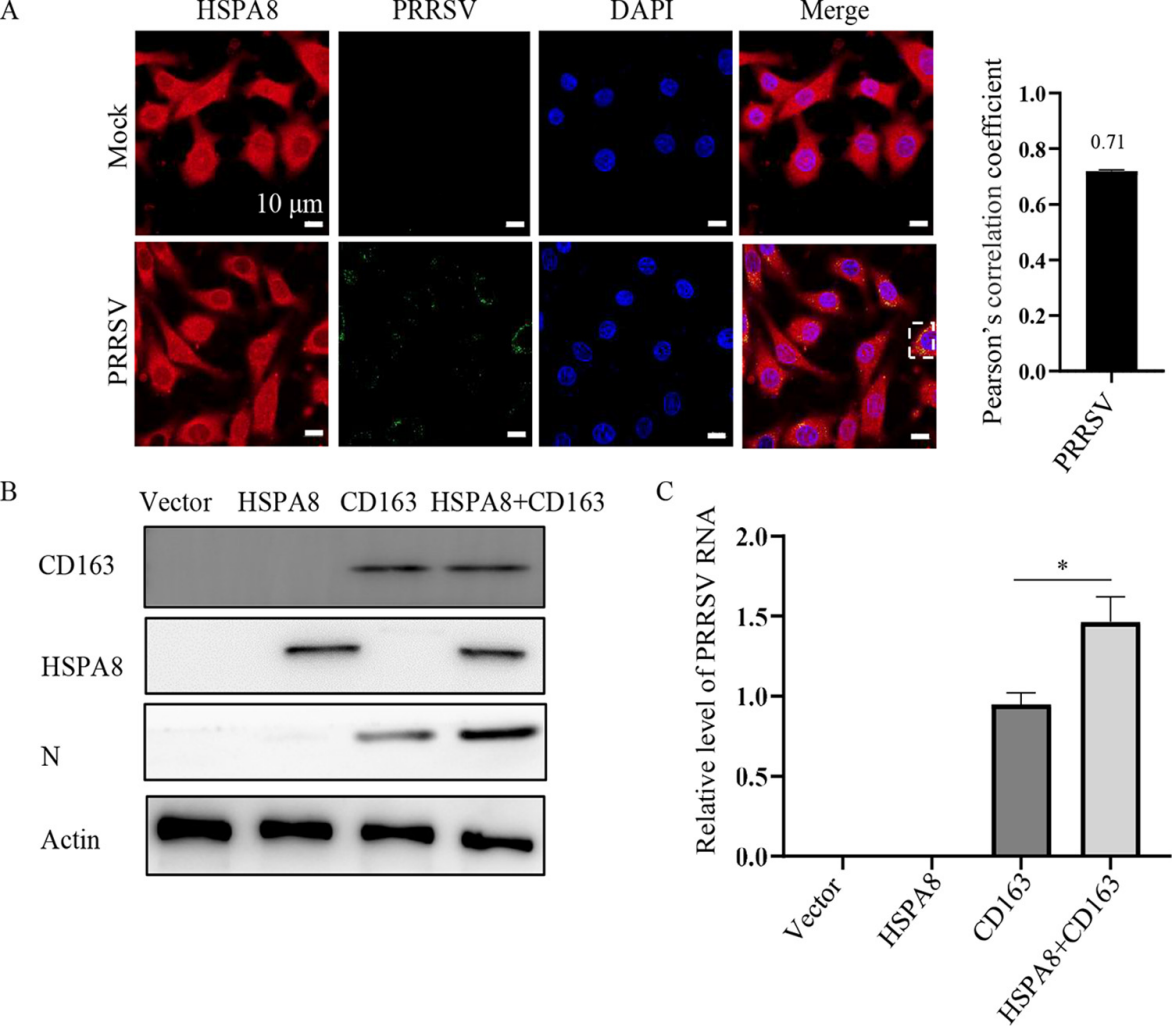
HSPA8 contributes to PRRSV infection along with CD163. (A) HSPA8 co-localized with internalized PRRSV in BHK-21 cells. The cells were inoculated with PRRSV (10 MOI) at 37°C for 0.5 h, and fixed with 4% PFA, permeabilized with 0.1% Triton X-100 and stained with anti-HSPA8 pAbs (catalog no. 10654-1-AP; red) and anti-PRRSV GP5 MAb (green). The cell nuclei were stained with DAPI (blue). Images were acquired on the Zeiss confocal microscope. The co-localization was assessed by determination of Pearson’s correlation coefficient. Scale bars, 10 µm. (B-C) HSPA8 contributed to PRRSV infection. BHK-21 cells were transfected with HSPA8-Myc and/or CD163 for 36 h and then infected with PRRSV (1 MOI) for 24 h. The cells were subjected to IB using anti-Myc MAb, anti-CD163 pAbs, anti-actin MAb, and anti-PRRSV N MAb (B). PRRSV RNA abundance was determined by RT-qPCR (C). Data represent means ± SD from three independent experiments. *, *P* < 0.05.

It is well-established that CD163 is an indispensable receptor for PRRSV infection (33, 34). As HSPA8 was shown to be involved in PRRSV attachment and internalization in the current study, we considered the involvement of CD163 and HSPA8 in PRRSV infection. We chose baby hamster kidney (BHK)-21 cells to distinguish their individual contribution to the viral infection. BHK-21 cells are refractory to PRRSV infection, while they are susceptible to viral infection with CD163 expression (35). As shown in Fig. 9A, PRRSV virions were internalized in BHK-21 cells and its co-localization with endogenous HSPA8 was observed (Pearson’s correlation coefficient >0.5). Interestingly, endogenous and over-expressed HSPA8 alone in BHK-21 cells was not sufficient to support PRRSV infection (Fig. 9B and C). However, co-expression of HSPA8 and CD163 contributed to PRRSV infection more than expression of CD163 alone. These results indicate that HSPA8 contributes to PRRSV infection along with CD163.

## DISCUSSION

PRRSV exploits various cell surface receptors/factors to infect host cells through its envelope proteins (36, 37). According to a previous model (38), PRRSV M protein initially binds to heparin sulfate (HS) on the cell surface (39). Subsequently, PRRSV M/GP5 heterodimer interacts with sialoadhesin (Sn), which mediates viral internalization (40). Upon internalization, PRRSV GP4 along with GP2, interacts with CD163, dominating viral tropism (27). Additionally, other factors on the host cell surface also facilitate PRRSV infection, such as CD151 (41), T-cell immunoglobulin and mucin domain (TIM) (42), dendritic cell-specific intercellular adhesion melecule-3-grabbing non-integrin (DC-SIGN; CD209) (43), syndecan-4 (SDC4) (44), and epidermal growth factor receptor (EGFR) (45). In this study, HSPA8 was found to interact with PRRSV GP4 and played an important role in viral attachment and internalization.

HSPA8 is a housekeeping chaperone, and responsible for maintaining protein homeostasis. In addition, HSPA8 has been shown to contribute to various viral infections (46). For instance, HSPA8 is involved in dengue virus (DENV) entry (47). HSPA8 also participates in Japanese encephalitis virus (JEV) endocytosis (48). Moreover, HSPA8 promotes Ebola virus (EBOV) minigenome replication in HEK-293T cells (49).

Here, we determined that HSPA8 interacted with PRRSV by binding to GP4 through its PB domain (Fig. 1 and 2). We further demonstrated that HSPA8 was involved in PRRSV infection dependent on its ATP hydrolytic activity (Fig. 3). In depth, HSPA8 was found to be expressed on the cell surface and cytoplasm of MARC-145 and CRL-2843-CD163 cells, and co-locate with PRRSV virions during attachment and internalization (Fig. 4, 6, and 7). Therefore, it attracted our attention on whether HSPA8 was involved in these processes. Both HSPA8 pAbs and soluble HSPA8 protein decreased PRRSV infection (Fig. 5, 7 and 8), showing that HSPA8 contributed to PRRSV attachment. Subsequently, we found that HSPA8 was involved in PRRSV internalization via CME (Fig. 6). As HSPA8 has been found to drive multiple steps of CME (27, 50), we tend to consider that PRRSV GP4 recruited and primed HSPA8 to reform clathrin-coated pits during CME. Intriguingly, HSPA8 alone wasn’t sufficient to support PRRSV infection as CD163 does (Fig. 9). We speculate that HSPA8 may work together with other receptors/factors during PRRSV infection.

**FIG 10:**
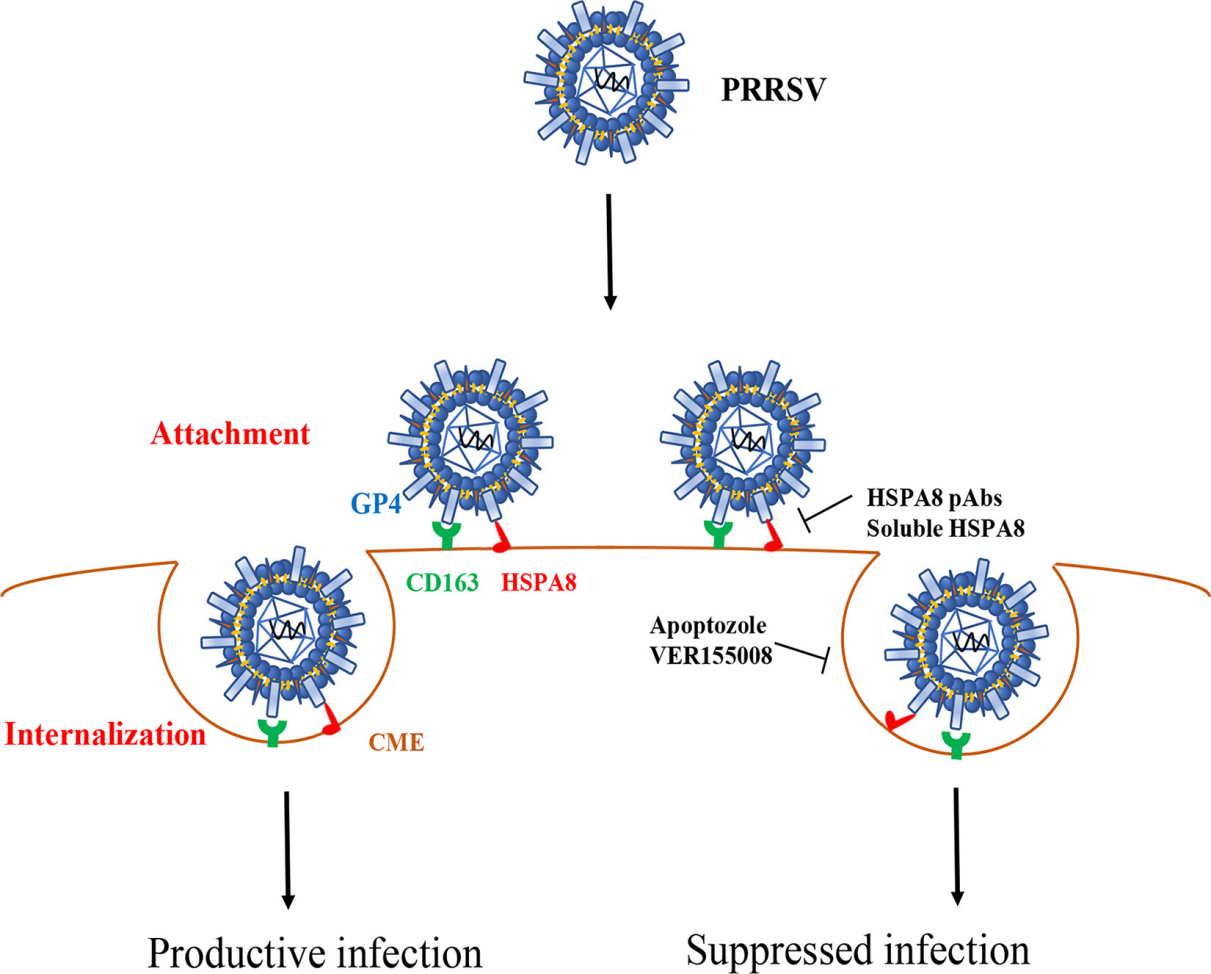
Model depicts the involvement of HSPA8 in PRRSV attachment and internalization. HSPA8 functions as a co-factor for PRRSV infection. In detail, HSPA8 is involved in PRRSV attachment and internalization via CME along with PRRSV indispensable receptor CD163. Interference with the interaction between HSPA8 and PRRSV on the cell surface inhibits viral infection in host cells by HSPA8 pAbs and soluble HSPA8 protein. In addition, PRRSV internalization is impaired by HSPA8 ATPase inhibitors VER155008 and apoptozole.

Based on the results stated above, we propose a model to depict the role of HSPA8 in PRRSV attachment and internalization (Fig. 10). In addition to CD163 and other receptors/factors, PRRSV GP4 binds to HSPA8 on the cell surface and then PRRSV is internalized into host cells via CME, where HSPA8 participates in as well.

In recent years, PRRSV keeps threatening global swine industry, and the development of vaccine and antiviral drugs is a critical and urgent task. Recent published studies have reported that CD163 antibodies strongly inhibit PRRSV infection via receptor blocking (51, 52), which may be developed into antiviral agents. In our study, HSPA8 pAbs also significantly suppressed PRRSV infection (Fig. 5, 7 and 8). Furthermore, chemical inhibitors exhibited a strong inhibitory effect on PRRSV infection (Fig. 3, 6 and 7). These inhibitors also took effect on other members in HSP family, like HSP90 and HSP70 (53), which were reported to facilitate PRRSV infection (53–55). Consequently, these results provide novel insights on antiviral drugs to restrain PRRSV infection. On the other hand, HSPs are regarded as important vaccine adjuvants and have been applied to cancer immunotherapy and viral control (56, 57). It has been reported that HSP70 fused with PRRSV GP3 and GP5 enhanced the immune responses and protective efficacy against virulent PRRSV challenge in pigs (58). These works infer that HSPA8 would be an attractive candidate vaccine adjuvant against PRRSV.

In conclusion, HSPA8 was identified as a co-factor for PRRSV attachment and internalization for the first time. Our findings are beneficial for the understanding of PRRSV infection, and provide insights into the development of novel effective vaccines and antiviral drugs for PRRSV.

## MATERIALS AND METHODS

### Cells and viruses.

MARC-145, BHK-21, and HEK-293T cells were maintained in Dulbecco’s modified Eagle’s medium (DMEM; catalog no. 12100; Solarbio, Beijing, China), supplemented with 10% fetal bovine serum (FBS; Gibco, Waltham, USA), and penicillin (100 U/mL; Solarbio) and streptomycin (100 μg/mL; Solarbio), at 37°C in a humidified incubator with a 5% CO_2_ atmosphere. CRL-2843-CD163 cells were stored in our laboratory, and routinely maintained in Roswell Park Memorial Institute 1640 medium (RPMI 1640; catalog no. 31800; Solarbio), supplemented with 10% FBS and antibiotics at 37°C in a 5% CO_2_ incubator. The PRRSV-2 strain BJ-4 (GenBank: AF331831) and PRRSV-1 strain GZ11-G1 (GenBank: KF001144) were kindly provided by Professor Hanchun Yang of China Agricultural University, China. The PRRSV-2 BJ-4 virions in GST pulldown assay and ELISA were gained according to a previous study (42). The PRRSV strain used in the experiments was PRRSV-2 strain BJ-4 unless otherwise stated.

### Antibodies, inhibitors, and reagents.

Rabbit anti-HSPA8 pAbs (catalog no. 10654-1-AP), mouse anti-HSPA8 monoclonal antibody (MAb; catalog no. 66442-1-Ig) and rabbit anti-ATP1A1 pAbs (catalog no. 14418-1-AP) were all purchased from Proteintech (Wuhan, China). Rabbit anti-mCherry pAbs (catalog no. GTX128508) were purchased from GeneTex (San Antonio, USA). Mouse anti-GAPDH MAb (catalog no. sc-47724) and mouse anti-tubulin MAb (catalog no. sc-23948) were purchased from Santa (Dallas, USA). Mouse anti-Myc MAb (catalog no. 2276), mouse anti-actin MAb (catalog no. 3700), rabbit anti-EEA1 pAbs (catalog no. 3288), and mouse anti-GST MAb (catalog no. 2624) were purchased from CST (Danvers, USA). Rabbit anti-GP5 pAbs (catalog no. bs-4504R) was purchased from BIOSS (Beijing, China). Horseradish peroxidase (HRP)-labeled goat anti-rabbit IgG antibody (catalog no. ab6721) and HRP-labeled goat anti-mouse IgG antibody (catalog no. ab6789) were purchased from Jackson (Cambridge, United Kingdom). Alexa Fluor 488-goat anti-mouse antibody (catalog no. A-11029), Alexa Fluor 647-goat anti-mouse antibody (catalog no. A-21235), Alexa Fluor 488-goat anti-rabbit antibody (catalog no. A-11008) and Alexa Fluor 647-goat anti-rabbit antibody (catalog no. A-21245) were purchased from Invitrogen (Carlsbad, USA). MAbs against PRRSV N protein and GP5 were kept in our laboratory (59). PAbs against PRRSV GP4 were a generous gift from Professor Zhiwen Xu of Sichuan Agricultural University, China.

VER155008 (catalog no. HY-10941) and apoptozole (catalog no. HY15098) were purchased from MCE (Shanghai, China).

Lipofectamine RNAiMAX transfection reagent (catalog no. 13778150) was purchased from Invitrogen. FuGENE transfection reagent was purchased from Promega (Madison, USA). Enhanced cell counting kit-8 (catalog no. C0042) and radioimmunoprecipitation assay (RIPA) (catalog no. C0042) were purchased from Beyotime (Shanghai, China).

### Plasmid constructs.

The gene fragment of PRRSV BJ-4 GP4 was synthesized and cloned to pLVX-mCherry-C1 by GENEWIZ (Suzhou, China). The recombinant plasmid expressed GP4 with a mCherry tag at the C-terminus (GP4-mCherry), and the plasmid pLVX-mCherry-C1 was used as control (mCherry). The full-length HSPA8 cDNA fragment was synthesized by GENEWIZ (Suzhou, China) according to monkey HSPA8 gene (GenBank: XM008021265). The full-length HSPA8, AB domain, and PB domain were sub-cloned into the pcDNA3.1(+)/myc-his A by GENEWIZ for mammalian cell expression. These plasmids expressed HSPA8-Myc, AB-Myc, and PB-Myc, respectively. The full-length HSPA8, AB domain, and PB domain were sub-cloned into the pGEX-4T-1 with GST tag by GENEWIZ for prokaryotic expression. The expressed proteins were named as HSPA8-GST, AB-GST, and PB-GST, respectively.

### mCherry-IP and MS analysis.

HEK-293T cells were transfected with the plasmids expressing mCherry and GP4-mCherry using FuGENE transfection reagent. At 36 h posttransfection, the cells were lysed with cell lysis buffer (catalog no. P0013; Beyotime) on ice for 20 min. Whole cell lysates (WCLs) were then centrifuged at 13,000 rpm for 12 min. Pierce Protein G Magnetic Beads (catalog no. 10004D; Thermo Fisher Scientific, Waltham, USA) were coupled with anti-mCherry antibody at 4°C overnight and then incubated with the supernatants. After incubation at 4°C overnight, the beads were washed six times with phosphate buffered solution (PBS). The associated proteins were analyzed through 12% sodium dodecyl sulfate-polyacrylamide gel electrophoresis (SDS-PAGE), and the protein bands in the gel were stained with sliver. The indicated protein bands were cut and applied to LC-MS/MS by Abace (Beijing, China). The top-ranked peptide matches were taken into consideration for protein identification.

### IP.

HEK-293T cells were co-transfected with mCherry or GP4-mCherry-expressed plasmids with HSPA8-Myc-expressed plasmid for 36 h. The cells were lysed in cell lysis buffer. The total cellular proteins were incubated with anti-mCherry antibody-coupled Pierce Protein G Magnetic Beads. Or HEK-293T cells were co-transfected with GP4-mCherry-expressed plasmid with HSPA8-Myc, AB-Myc, or PB-Myc-expressed plasmid, respectively, for 36 h. The total cellular proteins were incubated with Anti-Myc-Tag MAb (Agarose-conjugated, Abmart, Shanghai, China) at 4°C overnight according to the manufacturer’s protocol. The samples were washed with the lysis buffer or PBS for six times and detected by IB using the indicated antibodies.

### GST pulldown.

The recombinant proteins were expressed in Escherichia coli BL-21 (catalog no. CD601-02; TransGen, Beijing, China) and purified using BeaverBeads GSH (catalog no. 70601; Beaver, Suzhou, China) according to the manufacturer’s protocol. GST resins were incubated with purified GST-tagged proteins at 4°C for 2 h and then with PRRSV virions at 4°C overnight. After extensive washes with PBS for six times, the samples were eluted and subjected to IB with the indicated antibodies.

### ELISA.

ELISA was performed to evaluate the direct interaction between HSPA8 and PRRSV virions according to a previous study with minor modifications (60). Briefly, the 96-well ELISA plates (Corning, New York, USA) were coated with the purified recombinant GST-tagged proteins (0.1, 0.2, 0.4, 0.8 μM/well) in 0.05 M carbonate-bicarbonate buffer (pH 9.6) at 4°C for 12 h, and then blocked with 5% bovine serum albumin (BSA) at 37°C for 1 h. After three washes, the plates were incubated with PRRSV virions (10^8.1^ TCID_50_/mL in PBS, 100 μL/well) at room temperature (RT) for 2 h. To detect the interaction, anti-GP5 MAb at 1:3,000 was added to each well and incubated at 37°C for 1 h. The wells were washed three times and incubated with HRP-conjugated goat anti-mouse IgG antibody at 1:10,000 at 37°C for 1 h. The reactions were developed using 3,3′,5,5′-tetramethylbenzidine (TMB) and terminated with H_2_SO_4_. The optical density (OD) values were measured at 450 nm (OD_450_) using an ELISA microplate reader (BioTek, VT, USA). The experiments were performed in triplicate.

### RNA interference.

SiRNAs against HSPA8 and siRNA-negative control (siRNA-NC) were designed and synthesized by GenePharma (Shanghai, China). MARC-145 cells were transfected with the indicated siRNAs at a final concentration of 50 nM using Lipofectamine RNAiMAX according to the manufacturer’s instructions for 36 h. The transfected cells were applied for subsequent experiments. The indicated siRNAs are listed in Table 1.

**TABLE 1:** siRNAs used in this study

Target genes	5′–3′ (sense)	5′–3′ (antisense)
siRNA-491	GCUGGUCUCAAUGUACUUATT	UAAGUACAUUGAGACCAGCTT
siRNA-835	GGCCAGUAUUGAGAUCGAUTT	AUCGAUCUCAAUACUGGCCTT
siRNA-1596	GGGACAAGGUAUCAUCAAATT	UUUGAUGAUACCUUGUCCCTT
siRNA-NC	UUCUCCGAACGUGUCACGUTT	ACGUGACACGUUCGGAGAATT

### Cell viability detection.

Cell viability was detected by a cell counting kit-8 (CCK-8) according to the manufacturer’s instructions. Briefly, MARC-145 cells were seeded onto 96-well plates and treated with indicated inhibitors at different concentrations at 37°C for 24 h, or transfected with siRNAs at 37°C for 36 h. The CCK8 solution was added to each well, and then the plates were incubated at 37°C for 1.5 h. The OD_450_ was measured using a microplate reader (BioTek, VT, USA).

### Inhibitor treatments.

MARC-145 and CRL-2843-CD163 cells were treated with non-cytotoxic specific inhibitors or dimethyl sulfoxide (DMSO) for 1 or 24 h along with PRRSV at 37°C before subsequent experiments.

### pAb preparation.

To prepare mouse pAbs against HSPA8, the BALB/c mice of 4 ∼ 6 weeks old were injected with 50 μg purified recombinant HSPA8-GST or GST protein emulsified in Freund’s complete adjuvant or Freund’s incomplete adjuvant (catalog no. F5881 and F5506; Sigma, St. Louis, USA). After 2 weeks postimmunization, the serum was collected and inactivated at 56°C for 30 min. The experimental procedure was authorized and supervised by the Ethical and Animal Welfare Committee of Key Laboratory of Animal Immunology of the Ministry of Agriculture of China (permit no. LLSC410090).

### pAb inhibition assay.

The effects of HSPA8 pAbs on PRRSV infection were detected according to a previous study (61). MARC-145 cells on 24- or 96-well plates were incubated with HSPA8-GST pAbs in a 2-fold dilution series in DMEM (1:8-1:64) at 37°C for 1 h. GST pAbs were used in parallel as control. The cells were washed, and incubated with DMEM containing the corresponding antibodies and PRRSV-1 strain GZ11-G1 (1 multiplicity of infection, 1 MOI) or PRRSV-2 strain BJ-4 (0.1 MOI) at 4°C for 1 h. After three washes, the cells were again incubated with DMEM containing the corresponding antibodies at 37°C. At 24 hpi, the cells were harvested and subjected to detection. The effect on CRL-2843-CD163 cells was also tested with 0.3 MOI PRRSV-2 strain BJ-4 and corresponding antibodies in a 16-fold dilution.

### Protein inhibition assay.

Inhibition by recombinant HSPA8 protein *in vitro* was performed as a previous study (61). PRRSV-1 strain GZ11-G1 (1 MOI) or PRRSV-2 strain BJ-4 (0.1 MOI) was incubated with recombinant HSPA8-GST at different concentrations (0.5, 1, 2 μM), or recombinant GST protein at 2 μM at 37°C for 2 h. The MARC-145 cells cultured in 24- or 96-well plates were incubated with PRRSV-HSPA8 mixture at 37°C for 1 h. After washes, the cells were cultured in DMEM for another 24 h, and subjected to detection. For CRL-2843-CD163 cells, 0.3 MOI PRRSV-2 strain BJ-4 was inoculated, and the concentration of recombinant proteins was 2 μM.

### Transferrin uptake assay.

MARC-145 cells were grown on glass coverslips and transfected with siRNAs. After 36 h, the cells were incubated with transferrin (catalog no. 009-160-150; Jackson) at the final concentration of 5 μg/mL in DMEM for 0.5 h. The cells were washed three times with PBS and sodium citrate buffer (CBS; 0.1 M, pH 4.5) to remove extracellular transferrin, followed by PBS. The cells were then fixed with 4% paraformaldehyde (PFA) in PBS and subsequently permeabilized with 0.1% Triton X-100 in PBS. The internalized transferrin was examined by confocal microscopy.

### BHK-21 over-expression assay.

BHK-21 cells were transfected with HSPA8-Myc alone or with CD163 for 24 h, and then infected with PRRSV (1 MOI) for another 24 h. The plasmid expressing CD163 was kept in our laboratory (62). The cells were harvested for PRRSV N protein and RNA abundance detection.

### Quantitative real-time PCR (RT-qPCR).

Total RNA was extracted using TRIzol reagent (catalog no. 15596018; Invitrogen), and cDNA was produced by reverse transcription using a PrimeScript RT reagent kit (catalog no. RR037A; TaKaRa, Dalian, China) in accordance with the manufacturer’s instructions. The cDNAs from different samples were amplified by RT-qPCR to measure PRRSV open reading frame 7 (ORF7) abundance with GAPDH mRNA as endogenous control. The RT-qPCR was performed using SYBR green regent (catalog no. 4913850001; Roche, Basel, Switzerland) on LightCycler480 II (Roche, Basel, Switzerland) and programmed as follows: 94°C for 5 min (1 cycle), 94°C for 15 s and 60°C for 34 s (40 cycles). The primers were listed in Table 2. The fold change was calculated using the 2^−ΔΔCt^ method in technical and biological triplicates (63).

**TABLE 2:** Primers for RT-qPCR used in this study

Target genes	Sequence (5′–3′)	
	Sense	Antisense
PRRSV-ORF7	AAACCAGTCCAGAGGCAAGG	GCAAACTAAACTCCACAGTGTAA
Pig GAPDH	CCTTCCGTGTCCCTACTGCCAAC	GACGCCTGCTTCACCACCTTCT
Monkey GAPDH	GAAGGTGAAGGTCGGAGTCA	CATGTAAACCATGTAGTTGAGGTC

### IB.

The cellular proteins were separated by 8% or 12% SDS-PAGE, and then transferred onto polyvinylidene fluoride (PVDF) membranes (catalog no. 03010040001; Merck Millipore, Darmstadt, Germany). Next, the membranes were blocked by 5% skim milk in PBS with 0.05% Tween 20 (PBST), and incubated with the indicated primary antibodies at 4°C overnight. After six washes with PBST, the membranes were incubated with HRP-conjugated secondary antibodies at RT for 2 h. The protein bands were visualized using enhanced chemiluminescence reagent (catalog no. P2300; NCM, Suzhou, China).

### Immunofluorescence assay and confocal microscopy.

MARC-145, BHK-21, or CRL-2843-CD163 cells were grown in glass coverslips, fixed with 4% PFA for 15 min, permeabilized with 0.1% Triton X-100 or not in PBS for 5 min and blocked with 5% BSA blocking buffer (catalog no. SW3015; Solarbio). Nuclei were stained with 4, 6-diamidino-2-phenylindole (DAPI, catalog no. c0060; Solarbio) and observed via fluorescence microscopy (LSM800, Carl Zeiss AG, Oberkochen, Germany) with the confocal laser scanning set up (10×, 40× or 63× objective). Manders’ overlap coefficient (>0.6) and Pearson’s correlation coefficient (>0.5) are considered to represent the true degree of co-localization and interaction, respectively (21). Quantitative analyses of single channel fluorescence were performed using ImageJ software (64).

### PRRSV titration assay.

The transfected or treated cells were inoculated with PRRSV at an MOI of 0.1. The viruses not entering into the cells were then washed away. At 24 hpi, the progeny virus titers were measured by detecting TCID_50_ in MARC-145 cells according to Reed and Muench (65). In brief, the MARC-145 cells in 96-well plates were incubated with 0.1 mL of 10-fold serially diluted (10^−8^ to 10^−1^) samples. Four days later, cytopathic effects (CPEs) were observed using the inverted microscope (Axiovert 40, Carl Zeiss AG, Oberkochen, Germany). The number of wells with the cells showing CPEs was counted.

### Statistical analysis.

All data were presented as group means and standard deviations (SD), and analyzed by Student's *t* test using GraphPad Prism software (version 8.0). *P value* < 0.05 was considered to be statistically significant.
